# The Immune System Strikes Back: Cellular Immune Responses against Indoleamine 2,3-dioxygenase

**DOI:** 10.1371/journal.pone.0006910

**Published:** 2009-09-07

**Authors:** Rikke Bæk Sørensen, Linda Berge-Hansen, Niels Junker, Christina Aaen Hansen, Sine Reker Hadrup, Ton N. M. Schumacher, Inge Marie Svane, Jürgen C. Becker, Per thor Straten, Mads Hald Andersen

**Affiliations:** 1 Center for Cancer Immune Therapy (CCIT), Department of Hematology, Herlev University Hospital, Herlev, Denmark; 2 Division of Immunology, The Netherlands Cancer Institute, Amsterdam, The Netherlands; 3 Department of Dermatology, University of Würzburg, Würzburg, Germany; New York University School of Medicine, United States of America

## Abstract

**Background:**

The enzyme indoleamine 2,3-dioxygenase (IDO) exerts an well established immunosuppressive function in cancer. IDO is expressed within the tumor itself as well as in antigen-presenting cells in tumor-draining lymph nodes, where it promotes the establishment of peripheral immune tolerance to tumor antigens. In the present study, we tested the notion whether IDO itself may be subject to immune responses.

**Methods and Findings:**

The presence of naturally occurring IDO-specific CD8 T cells in cancer patients was determined by MHC/peptide stainings as well as ELISPOT. Antigen specific cytotoxic T lymphocytes (CTL) from the peripheral blood of cancer patients were cloned and expanded. The functional capacity of the established CTL clones was examined by chrome release assays. The study unveiled spontaneous cytotoxic T-cell reactivity against IDO in peripheral blood as well as in the tumor microenvironment of different cancer patients. We demonstrate that these IDO reactive T cells are indeed peptide specific, cytotoxic effector cells. Hence, IDO reactive T cells are able to recognize and kill tumor cells including directly isolated AML blasts as well as IDO-expressing dendritic cells, i.e. one of the major immune suppressive cell populations.

**Conclusion:**

IDO may serve as an important and widely applicable target for anti-cancer immunotherapeutic strategies. Furthermore, as emerging evidence suggests that IDO constitutes a significant counter-regulatory mechanism induced by pro-inflammatory signals, IDO-based immunotherapy holds the promise to boost anti-cancer immunotherapy in general.

## Introduction

Recent advances in the understanding of the interplay between cancer cells and cells of the immune system demonstrated the capacity of the immune system to recognize and destroy neoplastic cells; nevertheless, despite the fact that neoplastic transformation is associated with the expression of immunogenic antigens, the immune system often fails to respond effectively to these antigens. Thus, the immune system becomes tolerant towards these antigens [1]. There is a general consensus, that this acquired state of tolerance must be overcome for cancer immunotherapy to succeed. The biological role of Indoleamine 2,3-dioxygenase (IDO) in the immune system is still a subject of active investigation. For example, endogenous IDO has been implicated as one mechanism by which maternal tolerance toward the fetus is maintained [2]. Likewise, IDO can mediate suppression of T-cell immunity to MHC-mismatched liver allografts [3]. Furthermore, IDO control T-cell responses to autoimmune disorders, and regulates the severity of a variety of experimental autoimmune disorders [4], [5]. Thus, several lines of evidence indicate that IDO is a major component to maintain the homeostasis of the immune system which, however, also contributes to tumor-induced tolerance. The expression and activation of IDO creates a tolerogenic milieu in the tumor and the tumor-draining lymph nodes (LN) either via direct suppression of T cells by degradation of the essential amino acid tryptophan or via enhancement of local regulatory T-cell (Treg) -mediated immunosuppression. With respect to the former, some of the biological effects of IDO are mediated through local depletion of tryptophan, whereas others are mediated via immunomodulatory tryptophan metabolites [6], [7].

IDO can be expressed within the tumor by tumor cells as well as tumor stromal cells, where it inhibits the effector phase of immune responses. In this setting, IDO is believed to inhibit the effector phase of the immune response [8], [9]. In a murine model it was observed that tumor cells transfected with IDO become resistant to immune eradication, even in mice in which a fully protective immune response had been established by immunization [9]. Most importantly, in the clinical situation it was repeatedly observed, that expression of IDO in tumor cells is associated with an impaired prognosis [10], [11].

Additionally, IDO-expressing antigen-presenting cells (APC) are present in tumor-draining LN, where they are believed to create a tolerogenic microenvironment. Indeed, IDO-expressing CD19^+^ plasmacytoid dendritic cells (DC) isolated from tumor-draining LN mediate profound immune suppression and T-cell anergy *in vivo* [12]–[14]; plasmacytoid DC from normal LN and spleen do not express IDO. It should be noted that very few cells constitutively express IDO in normal lymphoid tissue except in the gut. This implies that the DC in tumor-draining LN, which constitutively express IDO must receive a stimulus which is related to the presence of the tumor. This stimulus is believed to be delivered by activated Tregs migrating from the tumor to the draining LN. Tregs have been shown to induce IDO via cell-surface expression of CTLA-4 [15]. The induction of IDO converts the tumor-draining LN from an immunizing into a tolerizing milieu. Indeed, when IDO^+^ DC are injected *in vivo*, they create suppression and anergy in antigen-specific T cells in the LN draining the injection site [14], [16]. Hence, IDO is a critical cellular factor contributing to immune suppressive and tolerogenic mechanism in cancer. Consequently, IDO has become a very attractive target for the design of new anticancer drugs and several IDO inhibitors are currently being investigated [17], [18]. However, to date the possibility to harness the immune system to target IDO-expressing cells has not been explored. This is particular surprising since IDO-expressing cells antagonizes the desired effects of other immunotherapeutic approaches and a combination of IDO- and tumor-targeting immunotherapies should be highly synergistic. In the present study, we provide evidence for the immunogenicity of IDO by demonstrating the presence of spontaneous cytotoxic immune responses against IDO-expressing cells in cancer patients.

## Methods

### Patients

Peripheral Blood Mononuclear Cells (PBMC) were collected from cancer patients (renal cell carcinoma, melanoma, and breast cancer) and healthy controls. Blood samples were drawn a minimum of four weeks after termination of any kind of anti-cancer therapy. The majority of renal cell carcinoma patients had previously been treated with IL2 and IFN-α, most melanoma patients had received high dose IL2 and IFN-α, while all breast cancer patients were pre-treated with several kinds of chemotherapy, (e.g. epirubicin, docetaxel, cabecitabine), trastuzumab, and/or endocrine therapy. PBMC were isolated using Lymphoprep separation, HLA-typed (Department of Clinical Immunology, University Hospital, Copenhagen, Denmark) and frozen in FCS with 10% DMSO. The protocol was approved by the Scientific Ethics Committee for The Capital Region of Denmark and conducted in accordance with the provisions of the Declaration of Helsinki. Written informed consent from the patients was obtained before study entry.

### Peptides

Epitopes from IDO were predicted using the “Database SYFPEITHI” [19] in combination with manual examination of the protein sequence for MHC class I anchor residues. Eleven synthetic 9mer and 10mer peptides were produced: IDO1 (IDO_54-62_; QLRERVEKL), IDO2 (IDO_164-172_; (FLVSLLVEI), IDO3 (IDO_195-203_; TLLKALLEI), IDO4 (IDO_41-49_; FIAKHLPDL), IDO5 (IDO_199-207_; ALLEIASCL), IDO6 (IDO_320-328_; VLSKGDGL), IDO7 (IDO_383-391_; DLMNFLKTV), IDO8 (IDO_275-283_; VLLGIQQTA), IDO9 (IDO_101-109_; KVLPRNIAV), IDO10 (IDO_61-70_; KLNMLSIDHL), and IDO11 (IDO_341-350_; SLRSYHLQIV). The peptides were dissolved in DMSO (final concentration 10 mM) or distilled water (final concentration 2 mM). The HLA-A2 high affinity binding epitope HIV-1 pol_476-484_ (ILKEPVHGV) was used as irrelevant control. The HLA-A2 restricted Epstein - Barr virus peptide EBV_BMLF1280-288_ (GLCTLVAML) was used as control.

### Assembly assay for peptide binding to MHC class I molecules

The binding affinity of the synthetic peptides (Genscript) to HLA-A2 molecules, metabolically labelled with [^35^S]-methionine, was measured in the assembly assay, as described previously [20], [21]. The assay is based on peptide-mediated stabilization of empty HLA-molecules released upon cell lysis, from the TAP-deficient cell line T2. Stably folded HLA-molecules were immune-precipitated by using the HLA class I specific, conformation dependent monoclonal antibody (mAb) W6/32 and separated by isoelectric focusing (IEF) gel electrophoresis. Major histocompatibility complex (MHC) heavy-chain bands were quantified using the ImageGauge Phosphoimager program (FUJI Photo Film, Carrolton, TX). The intensity of the band is directly related to the amount of peptide-bound class I MHC complex recovered during the assay. Subsequently, the extent of stabilization of HLA-A2 is directly related to the binding affinity of the added peptide. The recovery of HLA-A2 was measured in presence of 100, 10, 1, 0.1 and 0.01 µM of the relevant peptide. The C_50_ value was calculated for each peptide as the peptide concentration required for half maximal stabilization of HLA-A2.

### ELISPOT assay

The ELISPOT assay was used to quantify peptide epitope-specific IFN-γ releasing effector cells as described previously [22]. In some experiments PBMC were stimulated once *in vitro* with peptide prior to analysis as described [23] to extend the sensitivity of the assay. After 7 days in culture with peptide and 40 U/ml IL-2 (PeproTech), cells were counted and analyzed in IFN-γ ELISPOT. Briefly, nitrocellulose bottomed 96-well plates (MultiScreen MAIP N45; Millipore) were coated overnight with IFN-γ capture mAb (Mabtech). The wells were washed, blocked by X-vivo medium and the effector cells were added in duplicates at different cell concentrations, with or without 10 µM peptide. The plates were incubated overnight. The following day, medium was discarded and the wells were washed prior to addition of biotinylated secondary Ab (Mabtech). The plates were incubated at room temperature (RT) for 2 hours, washed, and Avidin-enzyme conjugate (AP-Avidin; Calbiochem/Invitrogen Life Technologies) was added to each well. Plates were incubated at RT for 1 hour and the enzyme substrate NBT/BCIP (Invitrogen Life Technologies) was added to each well and incubated at RT for 5–10 min. Upon the emergence of dark purple spots, the reaction was terminated by washing with tap water. The spots were counted using the ImmunoSpot Series 2.0 Analyzer (CTL Analyzers).

### Flow cytometry

For tetramer stainings, PBMC from cancer patients and healthy donors as well as tumor infiltrating lymphocytes (TIL) from cancer patients were stimulated once *in vitro* with peptide, or analysed directly *ex vivo*. The CD8 T cells were isolated from PBMC using the Dynal CD8 negative isolation kit (Dynal Biotech) at day 7. The resulting T-cell cultures were stained with PE coupled tetramers, followed by antibody staining with an appropriate combination of the fluorochrome-coupled mAbs: CD8-allophycocyanin/APC-Cy7, CD3-FITC, CD45RO-FITC, CD45RA-PE-Cy5 and CD28-allophycocyanin (BD Bioscience). For comparison, cells were stained with appropriate isotype controls. Tetramers were prepared using the MHC-peptide exchange technology [24] as described in [25]. Tetramer stainings were performed in PBS +2% FCS, for 15 min., RT, in the dark, whereas antibody stainings were performed in PBS +2% FCS, for 20 min., 4°C, in the dark. The MHC tetramer complexes used were: HLA-A2/IDO5 (ALLEIASCL) and HLA-A2/HIV-1 pol_476–484_ (ILKEPVHGV). The samples were analyzed on BD FACS Aria, using DIVA software (BD Biosciences).

Cancer cells were examined for HLA-A2 expression using flow cytometry. Cells were stained with a fluorochrome-coupled HLA-A2 mAb (BD Bioscience). For comparison, cells were stained with an isotype matched control. The samples were analyzed on BD FACS Aria, using DIVA software (BD Biosciences). Assuming normality, HLA-A2 expression was given by a one-tailed two sampled t-test comparing MFI_HLA-A2_ and MFI_Isotype control_, where MFI is the Mean Fluorescence Intensity. For *p*-values <0.05 (significance level) cells were defined HLA-A2^+^. The fold of expression was defined as MFI_HLA-A2_/MFI_Isotype control_.

### Intracellular protein staining

PBMC, DC, and cancer cells were examined for intracellular IDO expression using flow cytometry. After fixation and permeabilization (Cytofix/Cytoperm, BD), cells were stained with anti-IDO antibody (Millipore Corporation). For comparison, cells were stained with an isotype matched control (Millipore Corporation). After incubation, cells were washed twice with Perm/Washbuffer (BD) before staining with a FITC-labeled secondary antibody (DAKO). The samples were analyzed on BD FACS Aria, using DIVA software (BD Biosciences). Assuming normality, intracellular IDO expression was given by a one-tailed two sampled t-test comparing MFI_IDO_ and MFI_Isotype control_, where MFI is the Mean Fluorescence Intensity. For *p*-values <0.05 (significance level) cells were defined IDO^+^. The fold of expression was defined as MFI_IDO_/MFI_Isotype control_.

### Dendritic cells (DC)

DC were generated from PBMC by adherence on culture dishes at 37°C for 60 min in RPMI-1640 enriched with 10% human AB serum. Adherent monocytes were cultured in RPMI-1640 supplemented with 10% human AB serum in the presence of IL-4 (1000 U/ml) and GM-CSF (250 U/ml) for 6 days. DC were matured by addition of IL-1β (1000 U/ml), IL-6 (1000 U/ml), TNF-α (1000 U/ml), and PGE_2_ (1 µg/ml).

### Establishment of antigen specific T- cell cultures and clones

PBMC from cancer patients were stimulated with irradiated (25 Gy), IDO5-loaded autologous DC (PBMC:DC ratio  = 3×10^6^∶3×10^5^), with 3 µg/ml β_2_m, 20 U/ml IL-12 (PeproTech), and 40 U/ml IL-7 (PeproTech). The cultures were stimulated every 10 days with irradiated autologous DC (2×) followed by irradiated PBMC (2×), with 40 U/ml IL-2 (PeproTech). After one month, growing cultures were tested for specificity for IDO5 in a standard ^51^Cr-release assay. PBMC from specific cultures were cloned by limiting dilution in the presence of 10^6^/ml irradiated (25 Gy) IDO5-loaded PBMC, and 120 U/ml IL-2. Every 3-4 days 50 µl fresh media were added containing IL-2 to a final concentration of 120 U/ml. Growing clones were expanded using IDO5-loaded PBMC (5×10^4^ cells/well) and 120 U/ml IL-2. After expansion the clones were tested for specificity and cytotoxic potential in a standard ^51^Cr-release assay.

### Cytotoxicity assay

Conventional ^51^Cr-release assays for CTL-mediated cytotoxicity was carried out as described elsewhere [26]. Target cells were T2-cells, *in vitro* generated autologous immature DC (iDC) and matured DC (mDC), *in vitro* generated allogeneic HLA-A2^+^ iDC and mDC, autologous *ex vivo* isolated monocytes, T cells and B cells (isolated using CD14^+^, CD3^+^ or CD19^+^ microbeads (MACS)), the natural killer target cell line K562, *ex vivo* enriched HLA-A2^+^ AML-blasts (B and T cells were depleted from the bone marrow of AML patients using CD19^+^ and CD3^+^ microbeads (MACS)), the breast cancer cell lines CAMA-1 and MDA-MB-231, as well as the colon cancer cell lines HCT-116 and SW480 (all available at the American Type Culture Collection (ATCC)), and FM55M [27]. Lysis were blocked using the HLA specific mAb W6/32 (2 µg/100 µl) [28]. In some assays, target cells were treated with 100 U/ml IFN-γ for 2 days.

### Down-regulation of IDO in cancer cells

Human SW480 cancer cells were transfected with indicated short hairpin RNA (ShRNA) plasmids obtained from SuperArray using FuGene6 (Roche) according to manufacturers instructions. Cells were lysed directly in LSB buffer (Sigma). The LSB lysates were boiled for 5 min. and loaded on 10% precast protein gels (BioRad). Proteins were electro transferred to a PVDF membrane (Millipore Corporation) by a semidry transfer method and probed with indicated antibodies according to manufacturers instructions. Blots were developed with the ECL system obtained from Amersham and a CCD camera (LAS-1000, Fujifilm). Following antibodies were used: anti-Cdk7 (MO-1) (Santa Cruz) and anti-IDO (Millipore Corporation).

### Statistical analysis

Statistical analysis was performed on data on intracellular IDO staining. Assuming normality, intracellular IDO expression was given by a one-tailed two sampled t-test comparing MFI_IDO_ and MFI_Isotype control_, where MFI is the Mean Fluorescence Intensity. For *p*-values <0.05 (significance level) cells were defined IDO^+^. The fold of expression was defined as MFI_IDO_/MFI_Isotype control_. HLA-A2 expression was determined using the same approach.

## Results

### IDO-derived HLA-A2-restricted T-cell epitopes

**Figure 1:**
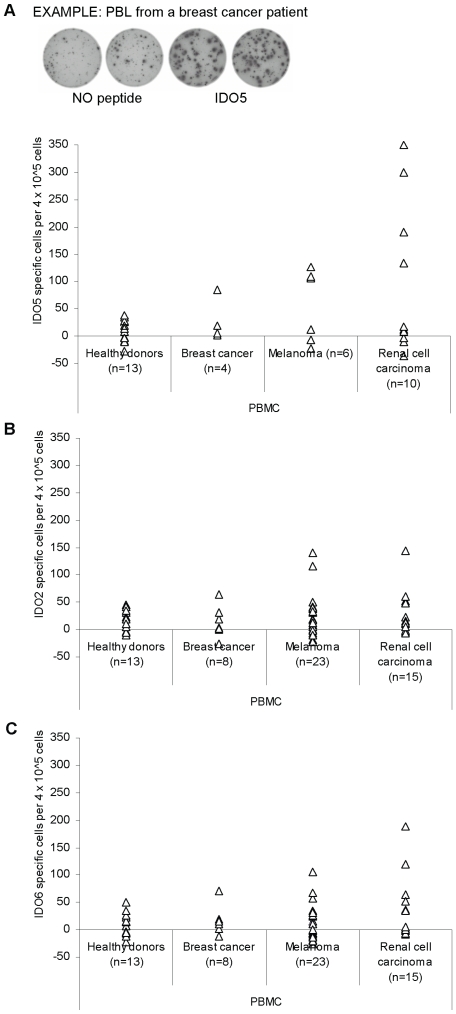
HLA-A2-restricted T-cell responses against IDO as measured by IFN-γ ELISPOT. PBMC from healthy donors, breast cancer patients, melanoma patients, and renal cell carcinoma patients were analyzed. All individuals were HLA-A2^+^. The peptides IDO5 (IDO_199-207_; ALLEIASCL) *(* *a* *)*, IDO2 (IDO_164-172_; FLVSLLVEI) *(* *b* *)*, and IDO6 (IDO_320-328_; VLSKGDGL) *(* *c* *),* were examined. T cells were stimulated once *in vitro* with peptide before being plated at 4×10^5^ cells per well in duplicates either without or with the relevant IDO peptide. The average number of IDO-specific spots (after subtraction of spots without added peptide) was calculated per 4×10^5^ PBMC for each patient (white triangle). *(* *a* *), Top,* Example of an ELISPOT response against IDO5 (IDO_199-207_; ALLEIASCL) in PBMC from a breast cancer patient.

Eleven IDO-derived peptides were selected from the main HLA-A2 specific anchor residues and subsequently synthesized [29]. Using the ELISPOT IFN-γ secretion assay, we then examined PBMC from cancer patients and healthy individuals for the presence of specific T-cell responses against these IDO-derived peptides. This approach has previously proved to be highly effective for identifying tumor specific cytotoxic T-lymphocytes (CTL) in cancer patients [22], [30], [31]. Thus, PBMC from HLA-A2^+^, late stage cancer patients (breast cancer, melanoma and renal cell carcinoma) were stimulated once with the different peptides *in vitro* before examination by ELISPOT. This procedure was chosen to extend the sensitivity of the ELISPOT as described [22], [32]. ELISPOT responses were detected against IDO2 (IDO_164-172_; FLVSLLVEI) (Fig. 1b), IDO6 (IDO_320-328_; VLSKGDGL) (Fig. 1c) and, especially, IDO5 (IDO_199-207_; ALLEIASCL) (Fig. 1a). To this end, PBMC from a larger number of cancer patients were examined for reactivity against IDO2 and IDO6, since these responses were less frequent compared to IDO5. As control, we examined PBMC from healthy individuals for reactivity against these three IDO derived peptides. No spontaneous responses could be detected against any of the IDO-derived peptides in any of the healthy controls. A BLAST search of the amino acid sequences of these peptides using the “NCBI database” showed that these motifs are only prevalent in the IDO protein.

### Detection of IDO-reactive HLA-A2-restricted T cells in cancer patients

**Figure 2:**
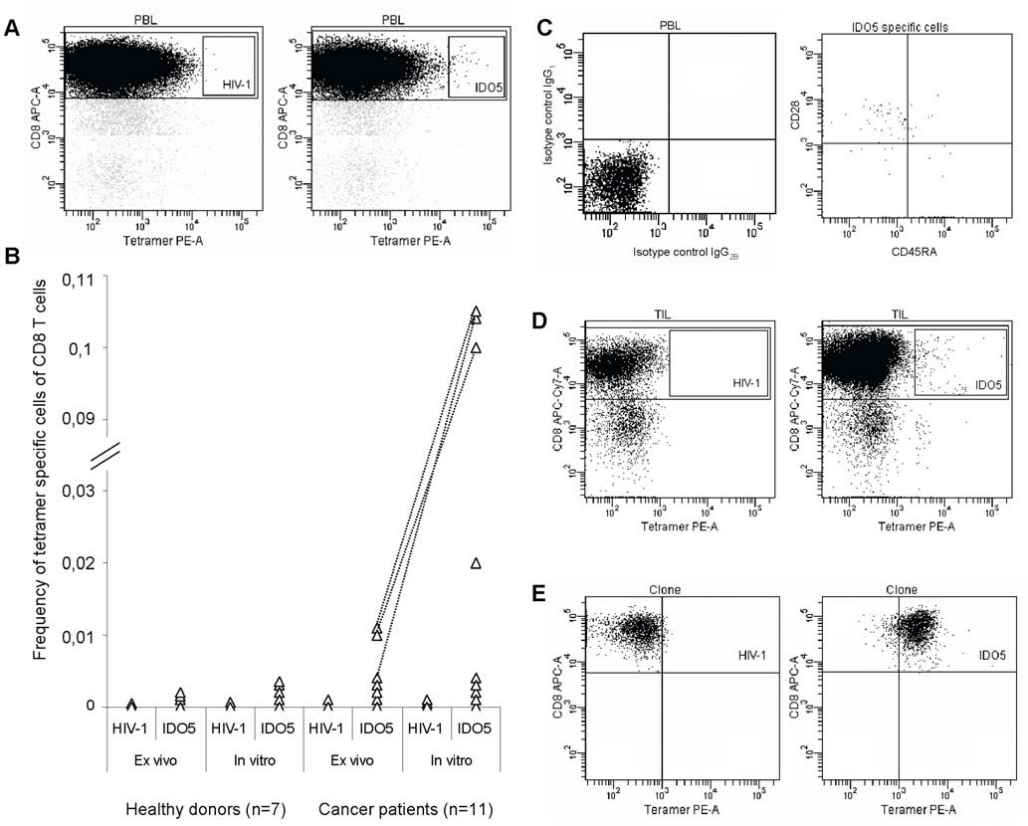
Tetramer analysis of IDO5-specific T cells. *(* *a* *),* An example of IDO5-specific, CD8 T-cell enriched PBMC from a renal cell carcinoma patient visualised by flow cytometry staining using the tetramer complex HLA-A2/IDO5-PE, and CD8-allophycocyanin after one *in vitro* stimulation with IDO5 peptide. As a negative control, cells were stained with the tetramer complex HLA-A2/HIV-1 pol_476–484_-PE, and CD8-allophycocyanin. *(* *b* *),* PBMC from healthy donors or from patients with breast cancer, melanoma cancer or renal cell carcinoma were stained with the tetramer complex HLA-A2/IDO5 or HLA-A2/HIV-1 pol_476–484_ and analyzed by flow cytometry either *ex vivo* or after one *in vitro* peptide stimulation. The dotted lines illustrate that IDO5-tetramer positive cells are detectable both *ex vivo* and *in vitro* in the same patients. *(* *c* *),* An example of CD45RA and CD28 phenotype analysis of IDO5-tetramer/CD8 gated cells from CD8 T-cell enriched PBMC from a renal cell carcinoma patient visualised *ex vivo* by flow cytometry. For comparison, the cells were stained with isotype matched controls *(* *d* *),* An example of an IL-2 expanded TIL culture from a melanoma patient visualised by flow cytometry staining using the tetramer complex HLA-A2/IDO5-PE, and CD8-APC-Cy7. As a negative control, the TIL were stained with the tetramer complex HLA-A2/HIV-1 pol_476–484_-PE, and CD8-APC-Cy7. *(* *e* *),* As a positive control of the IDO5 tetramer, an IDO5-specific T-cell clone (RBS35) was stained with the HLA-A2/HIV-1 pol_476–484_-PE and HLA-A2/IDO5-PE tetramers.

The apparently most immunogenic IDO-derived peptide, i.e. IDO5, was examined for its binding affinity to HLA-A2 by comparison with a HLA-A2 high affinity positive control epitope, i.e. HIV-1 pol_476-484_ (ILKEPVHGV), by the assembly assay (Table 1). For comparison, the HLA-A2 binding affinity of IDO2 and IDO6 were analyzed as well. C_50_ values were estimated for each peptide as the peptide concentration required for half maximal stabilization of HLA-A2. Notably, IDO5 bound HLA-A2 even better than the high-affinity control epitope, whereas IDO2 and IDO6 bound HLA-A2 with intermediate affinity compared to the control epitope (Table 1). The high binding affinity of IDO5 to HLA-A2 enabled us to make stable HLA-A2/IDO5 tetramers, which were used to detect IDO-reactive CTL by flow cytometry. This analysis clearly confirmed the presence of IDO5-reactive CD8 T cells in the blood of HLA-A2^+^ cancer patients (Fig. 2). Figure 2a illustrates an example of an IDO5-specific T cell response after *in vitro* stimulation in a renal cell carcinoma patient with an HIV-1 tetramer-complex used as control. While the frequency of IDO-reactive T cells are markedly increased by *in vitro* stimulation, IDO-reactive T cells were readily detectable *ex vivo* in selected patients (Fig. 2b): In the three patients with strongest responses after *in vitro* stimulation, a respective reactivity was also detected *ex vivo*. Overall, PBMC from 7 HLA-A2^+^ healthy individuals and 11 HLA-A2^+^ patients were analyzed. No IDO-reactive T cells could be detected in any of the healthy donors (Fig. 2b). The *ex vivo* stainings of IDO-reactive T cells showed that naturally occurring IDO5-specific T cells have a CD45RA^-^CD28^+^ central/effector memory phenotype [33]. An example of such an *ex vivo* phenotype staining of IDO5 tetramer gated cells is shown in figure 2c. As a comparison the sample were stained with isotype matched controls. Next, we examined the presence of IDO5-specific T cells in IL-2 treated TIL cultures from HLA-A2^+^ melanoma and head and neck cancer patients by tetramer stainings. As illustrated in figure 2d IDO5-specific T cells could readily be detected among the TIL. Overall, 4 of the 5 analyzed patients had detectable IDO5-specific T cells. The percentage of IDO5-specific T cells among the TIL ranged from 0.05 to 0.1% of the total number of CD8 T cells. Likewise, IDO5-specific T cells in TIL cultures from melanoma and head and neck cancer patients could be detected in ELISPOT (data not shown). To control the specificity of the HLA-A2/IDO5 tetramer we stained an IDO5-specific T-cell clone. The HLA-A2/IDO5 tetramer did efficiently stain the IDO5-specific T-cell clone, whereas the T-cell clone was not stained by the control HLA-A2/HIV-1 pol_476–484_ tetramer (Fig. 2e).

**Table 1:** HLA-A2 binding affinity of selected IDO-derived peptides.

Peptide	Sequence	C50 (mmol/L)*
HIV-1 pol _476-484_	ILKEPVHGV	0.3
IDO2 (IDO_164-172_)	FLVSLLVEI	30.0
IDO5 (IDO_199-207_)	ALLEIASCL	0.2
IDO6 (IDO_320-328_)	VLSKGDGL	3.5

* The C5 value is the peptide concentration required for half maximal stabilization of HLA-A2.

**Figure 3:**
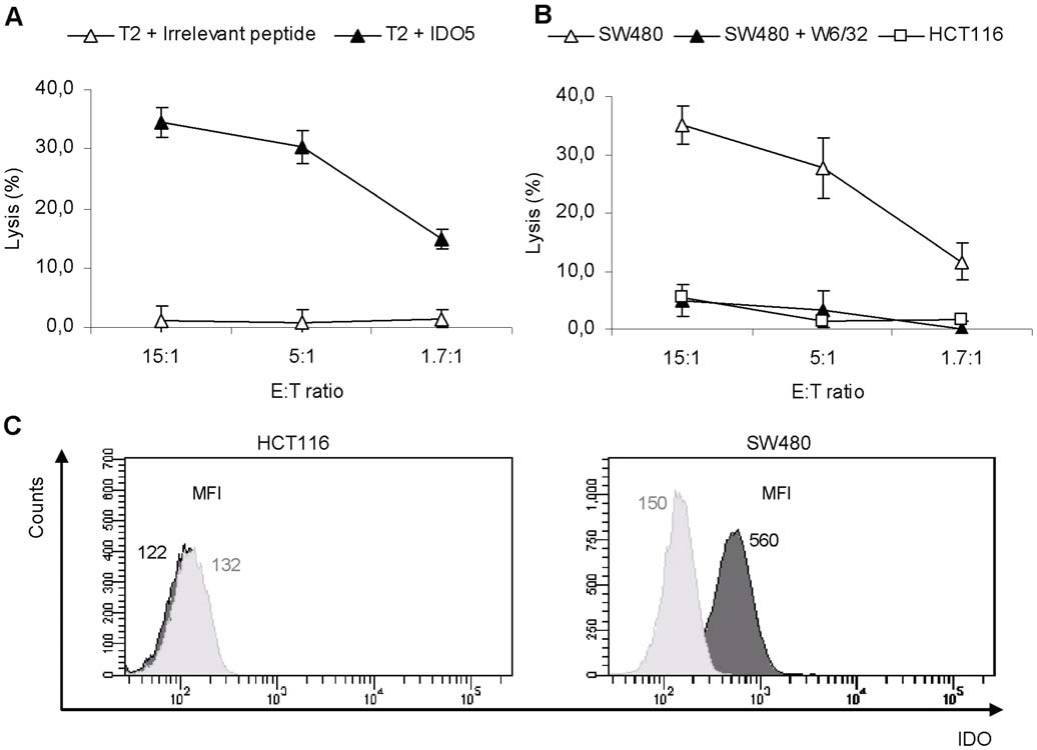
Specificity and functional capacity of IDO5-specific T-cells assayed by ^51^Cr-release assays. *(* *a* *),* Lysis of T2-cells pulsed with IDO5 peptide or an irrelevant peptide (the HLA-A2 high affinity binding epitope HIV-1 pol_476-484_ (ILKEPVHGV)) by a T-cell clone (RBS35). *(* *b* *)*, Specific lysis by RBS35 of the HLA-A2^+^/IDO^+^ colon cancer cell line SW480 without or with the addition of the HLA-class I specific antibody W6/32, and lysis of the HLA-A2^+^/IDO^-^colon cancer cell line HCT-116. All assays were performed in different E∶T ratios. *(* *c* *)* Histograms showing intracellular IDO expression in cancer cell lines. Data are representative of 3 experiments. Intracellular IDO expression was given by a one-tailed two sampled t-test comparing MFI_IDO_ (dark histograms) and MFI_Isotype control_ (light histograms), where MFI is the Mean Fluorescence Intensity. *Left:* HCT-116 (*p* = 0.300). *Right:* SW480 (*p* = 0.002).

Having identified patients hosting responses against the IDO5 peptide, we used PBMC from such patients to generate CTL bulk cultures against this peptide *in vitro*. PBMC were stimulated by autologous IDO5-pulsed DC. After four rounds of stimulation, the peptide specificity was tested in standard ^51^Cr-release assays. Bulk cultures were generated from three different cancer patients. Cells from two of these bulk cultures lysed TAP-deficient T2-cells pulsed with IDO5 peptide. To analyze the lytic capacity of IDO-specific T cells in more detail, CTL clones were established from one of these bulk cultures by limiting dilution cloning. After a short expansion period, the specificity of the growing clones was analyzed in standard ^51^Cr-release assays. Of thirty three T-cell clones displaying an IDO-specific lytic capacity, four clones were selected for further expansion due to a superior growth rate. A representative T-cell clone is depicted in figure 3a: the T-cell clone RBS35 effectively killed IDO5-pulsed T2-cells whereas T2-cells pulsed with a irrelevant peptide (HIV-1 pol_476-484_ (ILKEPVHGV)) were not lysed (Fig. 3a).

### Killing of tumor targets by IDO-specific T cells

Importantly, RBS35 killed not only peptide pulsed T2-cells but also the HLA-A2^+^/IDO^+^ colon cancer cell line SW480 with high efficacy (Fig. 3b). In contrast, RBS35 did not lyse the HLA-A2^+^/IDO^-^ colon cancer cell line HCT-116 (Fig. 3b). HLA-restriction of RBS35 was confirmed by blocking HLA-class I using the HLA specific mAb W6/32, which completely abolished lysis of the SW480 target cells (Fig. 3b). A number of cancer cell lines were examined for IDO expression by intracellular protein staining followed by FACS analysis [34]. The colon cancer cell line SW480, the melanoma cell line FM55M, the breast cancer cell lines CAMA-1 and MDA-MB231 as well as directly enriched AML-blasts were all IDO^+^. Only the colon cancer cell line HCT-116 was IDO^-^. Representative examples of IDO stainings are illustrated in histograms in figure 3c.

**Figure 4:**
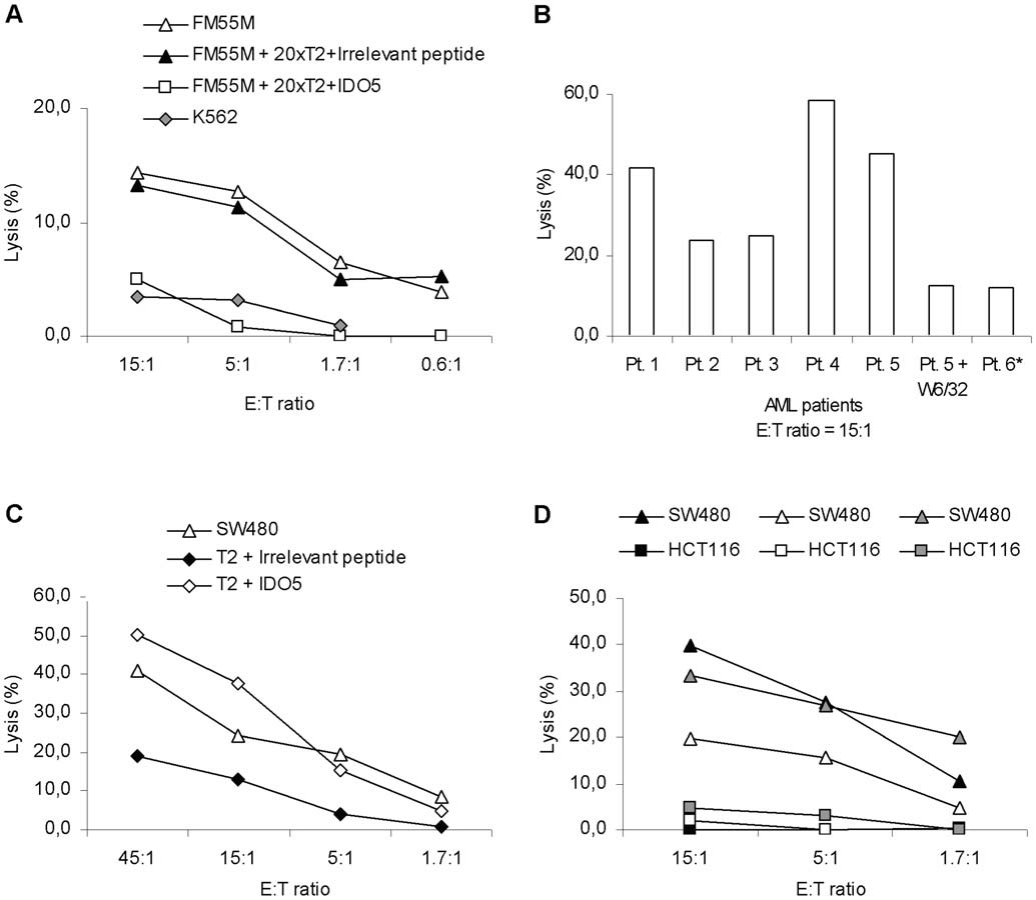
Specificity and functional capacity of IDO5-specific T cells assayed by ^51^Cr-release assays. *(* *a* *),* Lysis by the IDO5-specific T-cell clone (RBS35) of the HLA-A2^+^/IDO^+^ melanoma cell line FM55M without and with the addition of cold T2-cells pulsed with IDO5 peptide or an irrelevant peptide (HIV-1 pol_476-484_) (inhibitor to target ratio  = 20∶1), and NK cell activity of RBS35 examined using the natural killer cell line K562 as target cells. *(* *b* *),* Lysis by RBS35 of AML-blasts enriched from 5 HLA-A2^+^ AML patients and 1 HLA-A2^-^ AML patient. B cells and T cells were depleted from the bone marrow of the AML patients using CD19^+^ and CD3^+^ microbeads, respectively. The highly enriched AML-blasts were used as target cells with or without the addition of the HLA-class I specific antibody W6/32. *(* *c* *),* Lysis of T2-cells pulsed with IDO5 peptide or an irrelevant peptide (HIV-1 pol_476-484_), and lysis of the HLA-A2^+^/IDO^+^ colon cancer cell line SW480 by an IDO5-specific T-cell bulk culture. *(* *d* *),* Lysis of the HLA-A2^+^/IDO^+^ colon cancer cell line SW480 and HLA-A2^+^/IDO^-^ colon cancer cell line HCT-116 by three different IDO5-specific T-cell clones (RBS26 (*white triangle*), RBS31 (*black triangle*), RBS46 (*grey triangle*)) assayed by ^51^Cr-release assay. All assays were performed in different E∶T ratios.

Next, we demonstrated that the HLA-A2^+^/IDO^+^ melanoma cell line FM55M was killed by RBS35 (Fig. 4a). Cold targeted inhibition assays using unlabeled T2-cells pulsed with the IDO5 (10 µM) peptide confirmed the HLA-A2/peptide-specificity of the killing: The addition of cold (unlabeled) IDO5-pulsed T2-cells completely abrogated the killing of FM55M melanoma cells, whereas the addition of cold T2-cells pulsed with an irrelevant peptide (HIV-1 pol_476-484_) did not have an effect on the killing of FM55M (Fig. 4a). No cytotoxicity was observed against the NK-cell target cell line K562 (Fig. 4a).

Furthermore, we tested the ability of RBS35 to lyse human AML-blasts enriched directly *ex vivo* from the bone-marrow of AML patients. For this purpose, we depleted T cells (CD3^+^) and B cells (CD19^+^) from the bone marrow of AML patients; the highly enriched AML-blasts (CD3^-^, CD19^-^) were subsequently used as target cells in a ^51^Cr-release assay. We enriched AML blasts from six patients (5 HLA-A2^+^ patients and 1 HLA-A2^-^ patient). All AML blasts expressed IDO (data not shown). RBS35 efficiently lysed the HLA-A2^+^ leukemia cells in an HLA-dependent manner, while HLA-A2^-^ leukemia cells were not lysed (Fig. 4b). We did not find a correlation between the amount of IDO expression in the AML blasts and the percentage of lysis (data not shown).

To illustrate the representative killing of tumor targets by RBS35 the killing of SW480 by a polyclonal, IDO5-specific bulk culture as well as by three other T-cell clones (RBS26, RBS31, RBS46) are shown in figure 4c and figure 4d. The T cell clones had distinct TCR as verified by TCR Clonotype Mapping (data not shown). Similar to RBS35, none of the clones (RBS26, RBS31, RBS46) lysed the HLA-A2^+^/IDO^-^ colon cancer cell line HCT-116 (Fig. 4d).

**Figure 5:**
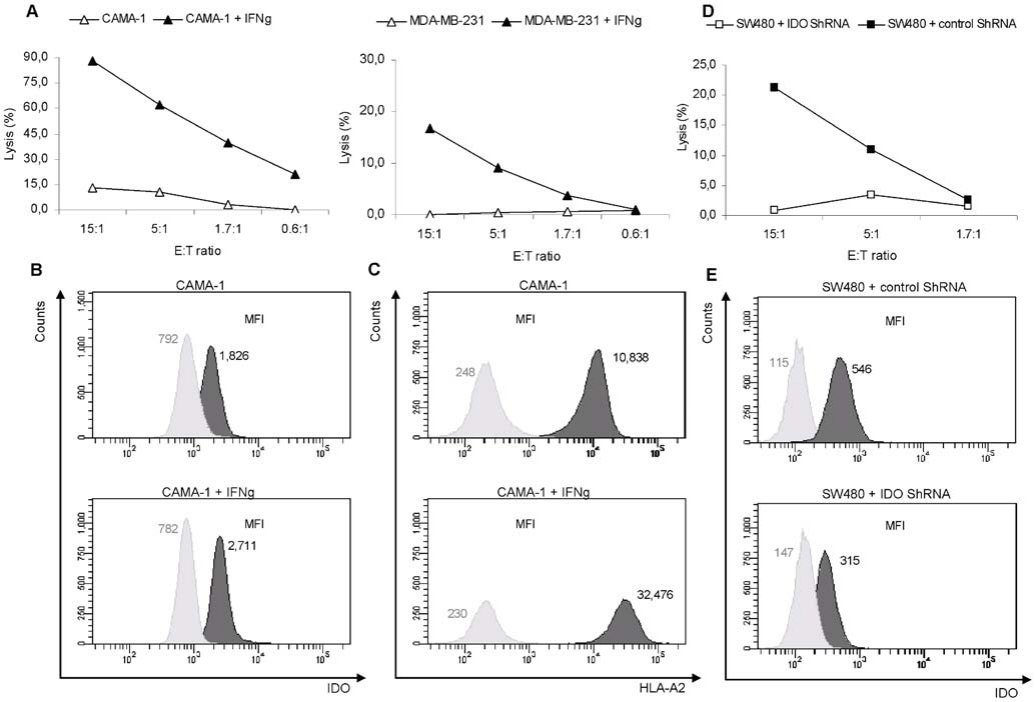
Functional capacity of IDO5-specific T cells to kill cancer cell lines treated with IFN-γ for up-regulation of IDO, or treated with IDO ShRNA for down-regulation of IDO. *(* *a* *),* Lysis by the IDO5-specific T-cell clone (RBS35) of the HLA-A2^+^ breast cancer cell lines CAMA-1 *(right)* and MDA-MB-231 *(left)* before and after IFN-γ treatment. *(* *b* *)*, Histograms showing intracellular IDO expression in CAMA-1 before and after IFN-γ treatment. Data are representative of 3 experiments. Intracellular IDO expression was given by a one-tailed two sampled t-test comparing MFI_IDO_ (dark histograms) and MFI_Isotype control_ (light histograms), where MFI is the Mean Fluorescence Intensity. The fold of expression was defined as MFI_IDO_/MFI_Isotype control_. *Top:* CAMA-1 (*p* = 0.020 and MFI_IDO_/MFI_Isotype control_  = 2.3). *Bottom:* CAMA-1 + IFN-γ treatment (*p* = 0.004 and MFI_IDO_/MFI_Isotype control_  = 3.5). *(* *c* *),* Histograms showing HLA-A2 expression in CAMA-1 before and after IFN-γ treatment. Data are representative of 3 experiments. HLA-A2 expression was given by a one-tailed two sampled t-test comparing MFI_HLA-A2_ (dark histograms) and MFI_Isotype control_ (light histograms). The fold of expression was defined as MFI_HLA-A2_/MFI_Isotype control_. *Top:* CAMA-1 (*p* = 0.004 and MFI_HLA-A2_/MFI_Isotype control_  = 43.7). *Bottom:* CAMA-1 + IFN-γ treatment (*p* = 0.002 and MFI_IDO_/MFI_HLA-A2_  = 141.2). *(* *d* *)*, Lysis of the colon cancer cell line SW480 transfected with IDO ShRNA for down-regulation of IDO protein expression by an IDO5-specific T-cell bulk culture. As a positive control, SW480 cells transfected with control ShRNA were used as target cells. All assays were performed in different E∶T ratios. *(* *e* *)*, Histograms showing intracellular IDO expression in SW480 transfected with control ShRNA (*p* = 0.001 and MFI_IDO_/MFI_Isotype control_  = 4.8) *(top)* and IDO ShRNA (*p* = 0.040 and MFI_IDO_/MFI_Isotype control_  = 2.1) *(bottom)*.

Finally, we examined the killing of the HLA-A2^+^ breast cancer cell lines CAMA-1 and MDA-MB-231. The CAMA-1 cell line was killed by RBS35, whereas MDA-MB-231 was not recognized by RBS35 (Fig. 5a). IFN-γ treatment increased the expression of IDO and HLA-A2 in both cell lines. This is exemplified for CAMA-1 in figure 5b and c. In agreement with this, IFN-γ treatment increased the killing by RBS35 of CAMA-1 and introduced killing of the MDA-MB-231 cells (Fig. 5a).

Additionally, using IDO ShRNA we down-regulated IDO protein expression in the human SW480 colon cancer cell line and thereby rescue these tumor cells from being killed by the polyclonal IDO-specific bulk culture, whereas cells transfected with irrelevant control ShRNA were killed (Fig. 5d). This down-regulation was visualized by intracellular protein stainings. These stainings confirmed that the use of IDO ShRNA reduced the level of IDO protein expression in the cells (Fig. 5e).

### Killing of dendritic cells by IDO-specific T cells

**Figure 6:**
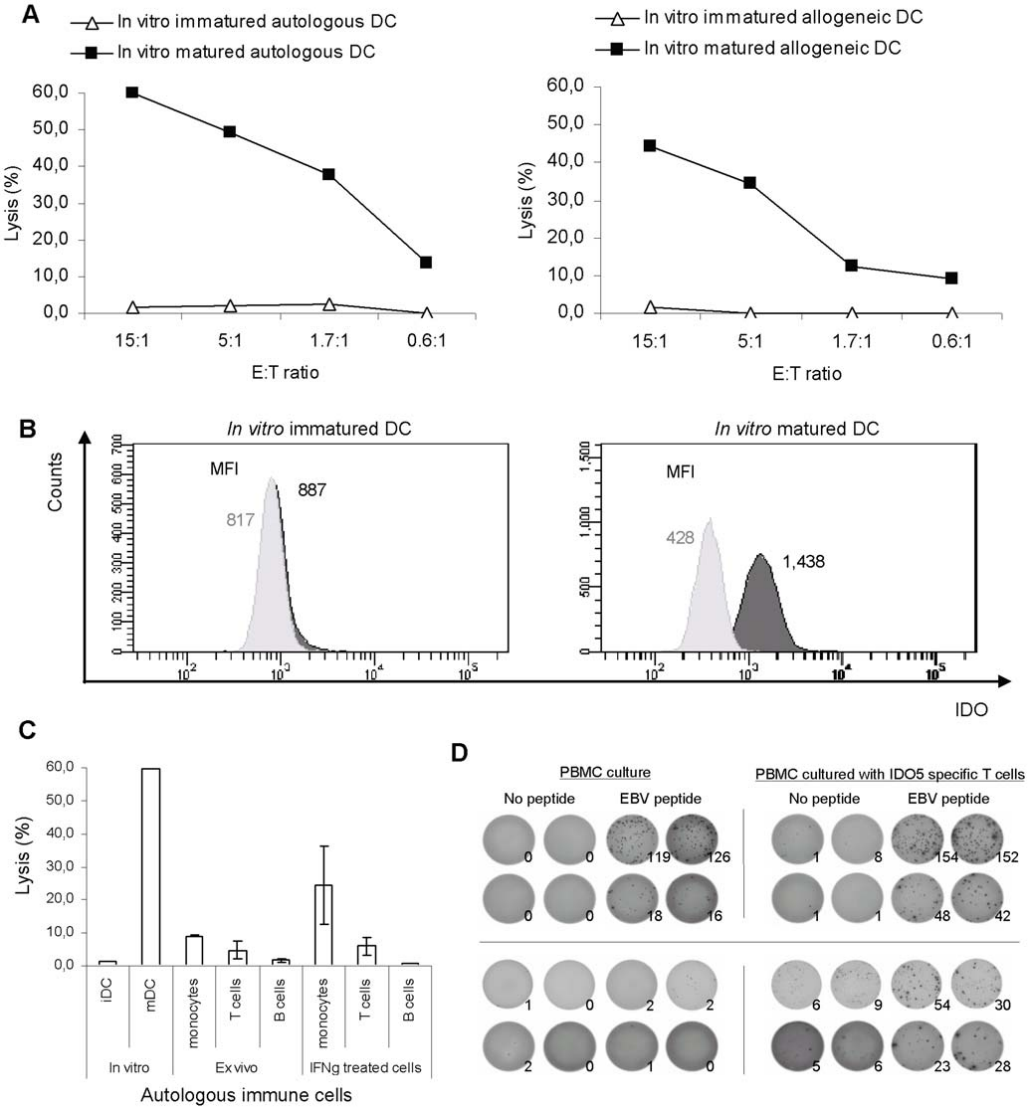
Functional capacity of an IDO5-specific T-cell clone (RBS35) to kill immune cells assayed by ^51^Cr-release assays. *(* *a* *)*, *Left:* Lysis of autologous *in vitro* immatured and matured DC. *Right:* Lysis of allogeneic HLA-A2^+^ *in vitro* immatured and matured DC. All assays were performed in different E∶T ratios. *(* *b* *),* Histograms showing intracellular IDO expression in DC. Data are representative of 3 experiments. Intracellular IDO expression was given by a one-tailed two sampled t-test comparing MFI_IDO_ (dark histograms) and MFI_Isotype control_ (light histograms), where MFI is the Mean Fluorescence Intensity. *Left: In vitro* immatured DC (*p* = 0.100). *Right: In vitro* matured DC (*p* = 0.001). *(* *c* *)*, Lysis of autologous CD14^+^ monocytes, CD3^+^ T cells and CD19^+^ B cells isolated directly *ex vivo* from IDO^+^ PBMC, and lysis of autologous CD14^+^ monocytes, CD3^+^ T cells and CD19^+^ B cells after IFN-γ treatment. As a control, we used *in vitro* generated autologous IDO^-^ immatured DC and IDO^+^ matured DC. *(* *d* *)*, Examples of HLA-A2 restricted T-cell responses against EBV BMLF1_280-288_ (GLCTLVAML) as measured by ELISPOT in PBMC from a breast cancer patient. Cultures of PBMC were treated with IFN-γ for 5 days with autologuous freshly isolated CD8 T-cells *(left)* or with autologous IDO5 specific T-cells (at a PBMC:IDO5 specific T-cell ratio of 300∶1) *(right)* before examination for reactivity against the HLA-A2 restricted epitope from EBV BMLF1_280-288_ (GLCTLVAML). Three different PBMC concentrations were examined; 1,5×10^5^ cells, 5×10^4^ cells (*two top rows*) and 10^4^ cells (*bottom two rows*).

IDO expression is not restricted to tumor and tumor stroma cells, but can also be induced in immune cells. Thus, as the next and even more important step we addressed the question whether IDO-expressing DC would also be susceptible to killing by IDO-reactive CTL. To test this notion, we generated autologous DC from the same donors from whom the CTL clones had been generated; the DC were matured by addition of a standard maturation cocktail consisting of IL-1β, IL-6, TNF-α, and PGE_2_ [35]. RBS35 effectively killed the *in vitro* matured DC (mDC). In contrast, autologous IDO^-^ immature DC (iDC) were not killed by RBS35 (Fig. 6a). Moreover, we examined the recognition of allogenic IDO^+^ mDC and IDO^-^ iDC from an HLA-A2^+^ donor. The allogenic mDC were killed by RBS35 whereas the allogenic IDO^-^ iDC were not killed (Fig. 6a). In figure 6b it is illustrated that mDC express IDO in contrast to iDC. Next, we tested the ability of RBS35 to lyse autologous monoctyes, T cells and B cells. For this purpose, we isolated CD14^+^ monocytes, CD3^+^ T cells and CD19^+^ B cells directly *ex vivo* from IDO^+^ PBMC. The isolated cells were subsequently used as target cells in a ^51^Cr-release assay. Autologous CD14^+^ monocytes, CD3^+^ T cells and CD19^+^ B cells were not lysed by RBS35 (Fig. 6c). IFN-γ treatment introduced killing of the CD14^+^ monocytes, but not of the T and B cells (Fig. 6c).

Finally, we sat up an *in vitro* model to examine if IDO specific T cells enhance immune responses by depleting IDO-expressing suppressive cells. Hence, cultures of PBMC, with and without IDO5 specific T cells, were treated with IFN-γ to increase the immune activity as well as IDO expression in the cultures. Five days later we examined the immune reactivity against the HLA-A2 restricted immunodominant epitope from EBV BMLF1_280-288_ (GLCTLVAML) in the cultures. Although the overall cell number was the same in the cultures the reactivity against the EBV peptide was higher in the cultures with IDO5-specific T cells (figure 6d). Next, we scrutinized if the addition of IDO specific T cells increased the immune reactivity to an extent that allowed detection of EBV responses in an ELISPOT with only 10^4^ PMBC; far below the normal detection limit. Indeed, we could detect a clear EBV response even at this low concentration of PBMC (Fig. 6d). As expected we could not detect any EBV response at this low cell concentration in the culture without IDO5 specific T cells (Fig. 6d).

## Discussion

IDO has a critical immunosuppressive function in cancer. In the present study, we set out to examine if IDO itself may serve as target for immune responses, which may be exploited for immune therapy. By following a ‘reverse immunology’ approach, we identified HLA-A2 peptides within the IDO protein to which spontaneous T-cell reactivity were detected in patients suffering from unrelated tumor types, i.e. melanoma, renal cell carcinoma and breast cancer but not in healthy individuals. These naturally occurring T-cell responses in cancer patients could be readily visualized by flow cytometry using HLA/peptide tetramers after *in vitro* stimulation but even more remarkable in direct *ex vivo* assays. In this regard, it should be noted that with a few exceptions it is not possible to detect conventional tumor associated antigen specific T cells in PBMC from cancer patients directly *ex vivo*, i.e. without any *in vitro* steps to expand or enrich these cells [32]. Thus, in some cancer patients IDO-specific T cells are present in relative high frequencies. We further reveal that IDO-specific T cells readily can be detected in the tumor microenvironment in lesions from both head and neck cancers and melanoma using tetramer stainings as well as ELISPOT. The combined detection of IDO-specific T cells in blood and tumor lesions indicate that these cells are capable of circulating and homing to the effector site. This is a significant finding, since several clinical reports have indicated the existence of a functional dissociation between local and systemic anti-cancer T-cell responses [36], [37].

Furthermore, we confirm that IDO-reactive T cells are indeed peptide specific, cytotoxic effector cells. Hence, an important issue concerning translation of our findings to a clinical setting lies in demonstrating the cytotoxic capacity of IDO-specific T cells against clinically relevant target cells. In this regard, IDO-specific T cells effectively lysed IDO^+^ cancer cell lines of different origin, such as melanoma, colon carcinoma, and breast cancer. Most importantly, leukaemia cells enriched directly from AML patients were killed *ex vivo* by IDO-specific T cells. IDO expression in blasts of AML patients have been correlated to significantly shorten overall and relapse-free survival [38]. The presence of spontaneous T-cell responses against IDO-derived peptide epitopes in PBMC from patients suffering from unrelated cancer types as well as the killing of cancer cells of different origin by IDO-specific T cells underline the immunotherapeutic potential of IDO. The induction of IDO expression after in *vitro* maturation of DC might be a major problem and an explanation for the lack of success of DC-based immunotherapy. However, even more distinctive was our finding that IDO-specific CTL recognize and kill IDO^+^ *in vitro* matured DC; hence, IDO-specific T cells are indeed able to kill immune suppressive cells. It is well described that IDO is up regulated in DC in tumor draining LN creating a tolerogenic microenvironment. Furthermore, DC isolated from cancer patients has impaired functionality and possesses an altered phenotype compared to healthy individuals. Hence, IDO vaccination might restore the ability of DC in cancer patients to initiate and/or activate anti-cancer immune responses by killing immune competent DC.

Counter-regulatory responses are important in the immune system as they help to limit the intensity and extent of immune responses, which otherwise could cause dangerous damage to the host. However, with regard to anti-cancer immunotherapy counter-regulatory responses antagonize the ability to create an intense immune response against the tumor. Counter-regulation differs from tolerance in the sense that counter-regulation is a secondary event, elicited only in response to immune activation. By definition most anti-cancer immunotherapeutic strategies irrespective of their molecular targets aim at the induction of an immunological activation and inflammation. Virtually, within the limits of acceptable toxicity as much immune activation as possible is the goal; hence, counter-regulation is not desired. IDO may be highly relevant to the outcome of immunotherapy of cancer as an inflammation-induced counter-regulatory mechanism. Hence, IDO is known to be induced by both type I and II interferons, which are found at sites of immune activation and inflammation [39], [40]. In this regard, we demonstrated that that the susceptibility of tumor cells to killing by IDO-reactive T cells is increased by preincubation with IFN-γ although this increased recognition might also be due to higher expression of HLA on the surface after IFN-γ treatment. Furthermore, we included a model where the addition of IDO specific T cells to IFN-γ treated PBMC increased the immune reactivity towards EBV. Hence, our findings suggest that IDO-based immunotherapy would work synergistically with additional therapy that introduces inflammation at the site of the tumor. Additionally, we illustrate that down-regulation of IDO rescue tumor cells from being killed by IDO-specific T cells. In this regard, immunoselection of antigen-loss variants during immunotherapy have been demonstrated in several cases [36], [41]–[44]. Accordingly, down regulation of IDO during IDO-based immunotherapy might save cancer cells and immune suppressive cells for immune-mediated destruction by IDO-specific CTL. This should, however, lead to removal of local immune suppression within the tumor and/or tumor draining LN and thereby enabling circulating effector cells to function or to get activated.

The state of lymphopenia following high-dose chemotherapy appears to provide a window of enhanced responsiveness to immunotherapy [45]. Additionally, certain chemotherapeutic drugs may assist in breaking immune tolerance by preferentially inhibition of Tregs [46], [47]. Nonetheless, chemotherapy alone is never sufficient to break tolerance to tumors probably because the original tolerogenic mechanisms rapidly restore tolerance following each cycle of chemotherapy. In principle, therefore, chemotherapy might provide an environment in which IDO-based immunotherapy could have a synergistic effect on established tumors, by preventing the re-acquisition of tolerance. This is supported by the report that the IDO blocker 1-Methyl-Tryptophan (1-MT) work synergistically with different chemotherapy drugs in established murine cancers [17].

In conclusion, we demonstrate that IDO is a natural target for CTL in cancer patients. Thus, we describe that although IDO up regulation has an immune suppressive effect in cancer, the immune system – at least in some patients - finds a way to fight back; by the induction of IDO-specific CTL. The immune dysfunction in the draining LN due to local IDO expression in DC is not indicative of a generalized immune suppression in cancer patients. Subsequently, it may well be possible to strengthen the described natural immunity against IDO by active immunotherapy. Since IDO-expressing cells might antagonize the desired effects of other immunotherapeutic approaches targeting IDO-expressing cells by vaccination would consequently be highly synergistic with such therapeutic measures. Finally, the recent ability of genetically modifying lymphocytes has opened possibilities for the *in vitro* creation of specific lymphocytes with appropriate therapeutic properties. Hence, the IDO T-cell receptors isolated from the T-cell clones described in the current manuscript can be introduced into a patient's normal lymphocytes and administered to lymphodepleted patients as described for other antigens [45], [48]–[50].

## References

[1] Morris E, Hart D, Gao L, Tsallios A, Xue SA (2006). Generation of tumor-specific T-cell therapies.. Blood Rev.

[2] Munn DH, Zhou M, Attwood JT, Bondarev I, Conway SJ (1998). Prevention of allogeneic fetal rejection by tryptophan catabolism.. Science.

[3] Miki T, Sun H, Lee Y, Tandin A, Kovscek AM (2001). Blockade of tryptophan catabolism prevents spontaneous tolerogenicity of liver allografts.. Transplant Proc.

[4] Hayashi T, Beck L, Rossetto C, Gong X, Takikawa O (2004). Inhibition of experimental asthma by indoleamine 2,3-dioxygenase.. J Clin Invest.

[5] Grohmann U, Fallarino F, Bianchi R, Orabona C, Vacca C (2003). A defect in tryptophan catabolism impairs tolerance in nonobese diabetic mice.. J Exp Med.

[6] Platten M, Ho PP, Youssef S, Fontoura P, Garren H (2005). Treatment of autoimmune neuroinflammation with a synthetic tryptophan metabolite.. Science.

[7] Bauer TM, Jiga LP, Chuang JJ, Randazzo M, Opelz G (2005). Studying the immunosuppressive role of indoleamine 2,3-dioxygenase: tryptophan metabolites suppress rat allogeneic T-cell responses in vitro and in vivo.. Transpl Int.

[8] Zou W (2005). Immunosuppressive networks in the tumour environment and their therapeutic relevance.. Nat Rev Cancer.

[9] Uyttenhove C, Pilotte L, Theate I, Stroobant V, Colau D (2003). Evidence for a tumoral immune resistance mechanism based on tryptophan degradation by indoleamine 2,3-dioxygenase.. Nat Med.

[10] Okamoto A, Nikaido T, Ochiai K, Takakura S, Saito M (2005). Indoleamine 2,3-dioxygenase serves as a marker of poor prognosis in gene expression profiles of serous ovarian cancer cells.. Clin Cancer Res.

[11] Weinlich G, Murr C, Richardsen L, Winkler C, Fuchs D (2007). Decreased serum tryptophan concentration predicts poor prognosis in malignant melanoma patients.. Dermatology.

[12] Sharma MD, Baban B, Chandler P, Hou DY, Singh N (2007). Plasmacytoid dendritic cells from mouse tumor-draining lymph nodes directly activate mature Tregs via indoleamine 2,3-dioxygenase.. J Clin Invest.

[13] Munn DH, Sharma MD, Hou D, Baban B, Lee JR (2004). Expression of indoleamine 2,3-dioxygenase by plasmacytoid dendritic cells in tumor-draining lymph nodes.. J Clin Invest.

[14] Munn DH, Sharma MD, Baban B, Harding HP, Zhang Y (2005). GCN2 kinase in T cells mediates proliferative arrest and anergy induction in response to indoleamine 2,3-dioxygenase.. Immunity.

[15] Thebault P, Condamine T, Heslan M, Hill M, Bernard I (2007). Role of IFNgamma in allograft tolerance mediated by CD4+CD25+ regulatory T cells by induction of IDO in endothelial cells.. Am J Transplant.

[16] Baban B, Hansen AM, Chandler PR, Manlapat A, Bingaman A (2005). A minor population of splenic dendritic cells expressing CD19 mediates IDO-dependent T cell suppression via type I IFN signaling following B7 ligation.. Int Immunol.

[17] Muller AJ, DuHadaway JB, Donover PS, Sutanto-Ward E, Prendergast GC (2005). Inhibition of indoleamine 2,3-dioxygenase, an immunoregulatory target of the cancer suppression gene Bin1, potentiates cancer chemotherapy.. Nat Med.

[18] Lob S, Konigsrainer A, Schafer R, Rammensee HG, Opelz G (2008). Levo- but not dextro-1-methyl tryptophan abrogates the IDO activity of human dendritic cells.. Blood.

[19] Rammensee HG, Falk K, Roetzschke O (1995). MHC molecules as peptide receptors.. Curr Biol.

[20] Elvin J, Cerundolo V, Elliott T, Townsend A (1991). A quantitative assay of peptide-dependent class I assembly.. Eur J Immunol.

[21] Andersen MH, Sondergaard I, Zeuthen J, Elliott T, Haurum JS (1999). An assay for peptide binding to HLA-Cw*0102.. Tissue Antigens.

[22] Andersen MH, Pedersen LO, Becker JC, thor Straten P (2001). Identification of a Cytotoxic T Lymphocyte Response to the Apoptose Inhibitor Protein Survivin in Cancer Patients.. Cancer Res.

[23] McCutcheon M, Wehner N, Wensky A, Kushner M, Doan S (1997). A sensitive ELISPOT assay to detect low-frequency human T lymphocytes.. J Immunol Methods.

[24] Toebes M, Coccoris M, Bins A, Rodenko B, Gomez R (2006). Design and use of conditional MHC class I ligands.. Nat Med.

[25] Rodenko B, Toebes M, Hadrup SR, van Esch WJ, Molenaar AM (2006). Generation of peptide-MHC class I complexes through UV-mediated ligand exchange.. Nat Protoc.

[26] Andersen MH, Bonfill JE, Neisig A, Arsequell G, Sondergaard I (1999). Phosphorylated Peptides Can Be Transported by TAP Molecules, Presented by Class I MHC Molecules, and Recognized by Phosphopeptide-Specific CTL.. J Immunol.

[27] Pawelec G, Marsh SG (2006). ESTDAB: a collection of immunologically characterised melanoma cell lines and searchable databank.. Cancer Immunol Immunother.

[28] Schmidt SM, Schag K, Muller MR, Weck MM, Appel S (2003). Survivin is a shared tumor-associated antigen expressed in a broad variety of malignancies and recognized by specific cytotoxic T cells.. Blood.

[29] Andersen MH, Tan L, Sondergaard I, Zeuthen J, Elliott T (2000). Poor correspondence between predicted and experimental binding of peptides to class I MHC molecules.. Tissue Antigens.

[30] Scheibenbogen C, Sun Y, Keilholz U, Song M, Stevanovic S (2002). Identification of known and novel immunogenic T-cell epitopes from tumor antigens recognized by peripheral blood T cells from patients responding to IL-2-based treatment.. Int J Cancer.

[31] Herr W, Ranieri E, Gambotto A, Kierstead LS, Amoscato AA (1999). Identification of naturally processed and HLA-presented Epstein-Barr virus peptides recognized by CD4(+) or CD8(+) T lymphocytes from human blood.. Proc Natl Acad Sci U S A.

[32] Keilholz U, Weber J, Finke JH, Gabrilovich DI, Kast WM (2002). Immunologic monitoring of cancer vaccine therapy: results of a workshop sponsored by the Society for Biological Therapy.. J Immunother.

[33] Wills MR, Okecha G, Weekes MP, Gandhi MK, Sissons PJ (2002). Identification of naive or antigen-experienced human CD8(+) T cells by expression of costimulation and chemokine receptors: analysis of the human cytomegalovirus-specific CD8(+) T cell response.. J Immunol.

[34] Boasso A, Herbeuval JP, Hardy AW, Anderson SA, Dolan MJ (2007). HIV inhibits CD4+ T-cell proliferation by inducing indoleamine 2,3-dioxygenase in plasmacytoid dendritic cells.. Blood.

[35] Nguyen XD, Eichler H, Sucker A, Hofmann U, Schadendorf D (2002). Collection of autologous monocytes for dendritic cell vaccination therapy in metastatic melanoma patients.. Transfusion.

[36] Lee KH, Panelli MC, Kim CJ, Riker AI, Bettinotti MP (1998). Functional dissociation between local and systemic immune response during anti-melanoma peptide vaccination.. J Immunol.

[37] thor Straten P, Guldberg P, Grønbæk K, Zeuthen J, Becker JC (1999). In Situ T-Cell Responses against Melanoma Comprise High Numbers of Locally Expanded T-Cell Clonotypes.. J Immunol.

[38] Chamuleau ME, van de Loosdrecht AA, Hess CJ, Janssen JJ, Zevenbergen A (2008). High INDO (indoleamine 2,3-dioxygenase) mRNA level in blasts of acute myeloid leukemic patients predicts poor clinical outcome.. Haematologica.

[39] Popov A, Schultze JL (2007). IDO-expressing regulatory dendritic cells in cancer and chronic infection.. J Mol Med.

[40] Scheler M, Wenzel J, Tuting T, Takikawa O, Bieber T (2007). Indoleamine 2,3-dioxygenase (IDO): the antagonist of type I interferon-driven skin inflammation?. Am J Pathol.

[41] Riker A, Cormier J, Panelli M, Kammula U, Wang E (1999). Immune selection after antigen-specific immunotherapy of melanoma.. Surgery.

[42] Pawelec G, Zeuthen J, Kiessling R (1997). Escape from host-antitumor immunity.. Crit Rev Oncog.

[43] Lehmann F, Marchand M, Hainaut P, Pouillart P, Sastre X (1995). Differences in the antigens recognized by cytolytic T cells on two successive metastases of a melanoma patient are consistent with immune selection.. Eur J Immunol.

[44] Wang Z, Seliger B, Mike N, Momburg F, Knuth A (1998). Molecular analysis of the HLA-A2 antigen loss by melanoma cells SK-MEL- 29.1.22 and SK-MEL-29.1.29.. Cancer Res.

[45] Morgan RA, Dudley ME, Wunderlich JR, Hughes MS, Yang JC (2006). Cancer regression in patients after transfer of genetically engineered lymphocytes.. Science.

[46] Ghiringhelli F, Menard C, Puig PE, Ladoire S, Roux S (2007). Metronomic cyclophosphamide regimen selectively depletes CD4+CD25+ regulatory T cells and restores T and NK effector functions in end stage cancer patients.. Cancer Immunol Immunother.

[47] Lutsiak ME, Semnani RT, De Pascalis R, Kashmiri SV, Schlom J (2005). Inhibition of CD4(+)25+ T regulatory cell function implicated in enhanced immune response by low-dose cyclophosphamide.. Blood.

[48] Morgan RA, Dudley ME, Yu YY, Zheng Z, Robbins PF (2003). High efficiency TCR gene transfer into primary human lymphocytes affords avid recognition of melanoma tumor antigen glycoprotein 100 and does not alter the recognition of autologous melanoma antigens.. J Immunol.

[49] Kuball J, Dossett ML, Wolfl M, Ho WY, Voss RH (2007). Facilitating matched pairing and expression of TCR chains introduced into human T cells.. Blood.

[50] Kuball J, Schmitz FW, Voss RH, Ferreira EA, Engel R (2005). Cooperation of human tumor-reactive CD4+ and CD8+ T cells after redirection of their specificity by a high-affinity p53A2.1-specific TCR.. Immunity.

